# First General Annual Report of the Dental Department

**Published:** 1915-11

**Authors:** George B. Hayes

**Affiliations:** Chn. Dentiste, D. F. M. P. Chief Dental Surgeon


### FIRST GENERAL ANNUAL REPORT

### Of the Dental Department

*To the Medical Board of the American Ambulance, for the Year September* 1, 1914, *to September*, 1915.

BY GEORGE B. HAYES, A.B., D.D.S.

**Chn. Dentiste, D. F. M. P. Chief Dental Surgeon**

I beg to submit the following summary report of the principal operations performed during the year just finished. This list is far from being complete, no mention being made of the great number of treatments necessarily involved in work of this kind, and I regret to say that a very considerable percentage of operations was not recorded owing, first, to the great and constant press of work which has kept steadily increasing, without relaxation, since the beginning; second, to the lack of early assistance and to insufficient organization, the quantity and character of the work having far surpassed all early conception.

### Installation.

The installation, from one dental chair alone at the start, has grown to number eight, with two dental laboratories.

The staff includes eight dental mechanics, three apprentices, two nurses, seven auxiliaries, and a typist, involving a total of thirty-two persons in the department.

At one time, in the month of May, over one-fifth (125 exactly) of the wounded in the Ambulance were under treatment for fractured maxillae, and in addition to the 560 beds in the Ambulance, the department was taking care of its own convalescent patients returning from eleven outside hospitals.

### Aims.

It has been our aim and effort to offer to each wounded man, at least before leaving the hospital, dental treatment, including prophylaxis of the mouth, extraction of broken down teeth, necessary fillings, and the substitution of artificial teeth whenever necessary for sufficient mastication or the correction of a visible deformity.

Several factors have interfered seriously with the complete realization of this ideal:

### Previous Neglect.

1. The extraordinary amount of work required, due to previous neglect—only a very small percentage of the wounded having ever visited a dentist except for the extraction of an aching tooth.

### Treatment of Personnel.

2. The considerable demand for treatment on the part of the personnel of the hospital, including the field ambulance and special cases referred to us particularly from hospitals without a dentist.

### Fractures.

3. And mainly the unprecedented number of fractures of the maxillae, the gravity of these factures involving daily and prolonged treatment; approximately one-half of this number being tardy arrivals, healed of infection, but, from lack of dental intervention, with vicious consolidation and deformation of the remaining parts, necessitating reduction either by section or by the application of gentle force with complicated apparati.

For the past eight months fully one-half of the time has been taken up by this class of treatment.

### Cards and Records.

Each patient is furnished an appointment card as soon as he is convalescent, offering him dental services. This card, in charge of the nurse, remains with his papers and indicates that either: (1) he has refused dental treatment; or (2) that on examination he required none; or (3) that his dental treatment is terminated; the dates of appointments indicating whether or not he is going regularly to the dental surgeon.

Records on separate cards are kept of all operations. When of sufficient interest, photographs are taken, as well as radiographs, impressions and plaster models made, which are filed together with the patient's history, diagnosis and record of treatment.

### Visits.

Visits to the wards are now confined to cases of suffering, of urgent treatment, or of special call for consultation.

### Expenses.

For the first two and a half months all the expense of furniture, instruments and supplies was borne by private contribution, and until the establishment of the laboratories, all of the mechanical work, for five months, was done free of expense to the Ambulance.

Several private individuals have contributed to the outlay for photographic records, and a very large quantity of supplies and perishable instruments has been given by no less than eight dental furnishing houses in America, thus reducing the actual expense far below the cost represented.

### Materials.

All materials used have been of best quality, and particular and extra effort has been made to give the work a durable nature. Except in a few instances, and at private expense, no gold was used up to the establishment of a "Gold and Platinum Fund."

### Laboratories.

An enormous increase in the output of work and in time saved was obtained by the establishment of the dental laboratory. The number of dental splints, of special apparati and of regular artificial dentures made (averaging one denture to every four men), has only been made possible by the assistance of these trained mechanics, and I can not do better than to insist on the important advantages gained by their mobilization for the purpose. Their services have been absolutely required for the accomplishment of this special work.

### Auxiliaries.

Another great step in saving of time has been the special appointment of one auxiliary nurse to each operator.

This service, just short of professional in character, requires usually several months of training to become efficient, but, when thoroughly developed, means a saving of one-third of the operator's time in actual practice.

At times monotonous and uninteresting, it has been rendered regularly with marked ability, devotion and patience. The same may be said of the work of keeping the records.

### Head Nurse.

The early appointment of a head nurse was a most useful and necessary expedient for the training and direction of the auxiliaries, for the supervision of dressings, the management of the wounded and for general order.

The Department was fortunate in the selection for this post and owes much to the ability and tact with which these duties were performed.

### X-Rays.

The X-Ray Department, while not equipped for the special work of dental photography, has rendered most valuable and indispensable service.

### History.

It is my belief that the distinction of being the first to have a dental surgeon on the medical board of a military hospital belongs to this ambulance.

It was therefore with a keen sense of responsibility to you and to the members of my chosen profession that I accepted a task wherein lay such exceptional opportunity of useful service and educational benefit.

My one aim and effort has been, aside from direct service to the wounded, to show the profession worthy of this new recognition and honor, and to demonstrate the necessity of its help. And it may well be a matter of extreme satisfaction to the Board that, thanks to their kind assistance, this department has been able to demonstrate the immense possibilities of dental surgery, especially, as in case of this ambulance, where the work has been so happily carried out in collaboration with the general surgeon.

On learning of a movement to organize some kind of aid to the wounded by the American Hospital, I presented myself and offered my services on August 4, 1914, just as the Committee were leaving to inspect the Lycee Pasteur. My appointment as dental surgeon soon followed and I began planning for the care of 150 wounded.

Within a fortnight enough funds were collected to warrant doubling the number of beds, and, feeling that alone I would be unable to care for that number, requested the appointment of my confrere, Dr. W. S. Davenport, and together we completed the installation of the dental department, bringing our own chairs, engines, instruments and supplies from a dental clinic which we had been conducting together for ten years.

There being no convalescents at the opening, we began by inspecting the mouths of the patients in bed, accomplishing general prophylactic work with disinfection, treating such teeth as threatened trouble and postponing further operations until the wounded might be able to go to the dental surgery.

The single operating chair was used alternately, with visits to the wards by both of us.

### Reputation.

The fact of the existence of our dental department soon became known, and the English army, without a single dentist, quartered at Villeneuve-Saint-Georges, early sougnt our assistance. Officers from the front on three days' leave of absence came to us for relief from dental troubles. Several telegrams were received requesting a dentist to be sent to the front.

### Lack of Dental Services.

Up to this time no other ambulance had made provision for a dental surgeon, although both the French and English soon after began the organization of similar departments, and we had numerous visitors to take note of our installation and methods of work.

Soon after the cases of "fractured jaws," as they came to be commonly called, began to arrive, and a new branch of dental surgery and dental "orthopedics" came into existence.

### Co-operation of General and Dental Surgeons.

A new line of work, of creative work, began, which has brought the general surgeon and the dental surgeon into a mutual co-operation and interdependence such as never before existed.

### New Branch of Service.

Fractures of the maxillae had been classified and their treatment defined, but no reference books could be found to record the treatment of the terrible devastation caused by the projectiles of modern warfare (trench fighting).

Almost every case presented fractures with loss of substance varying from small pieces to half or nearly the whole of the mandible or half the face.

### Difficulties.

The study then began of what remaining fragments should be removed and what saved; means devised to maintain these fragments in their normal position during the healing process, and later, devices for restoring lost parts and building up a skeleton or frame for the final grafts and plastic work of the surgeon.

At this time Professor Choquet joined us, bringing material aid from the Ecole Dentaire de Paris. It is to his initiative that we owe the first good photographic records and the early colored slides.

The scientific reports of these interesting and important cases will follow later, when they will have been duly classified, but in this general revision it may be fairly stated that the dental surgeons and dental mechanics have risen to the occasion, and the value of their work has been duly recognized.

### Results.

They have made it possible for a final plastic operation to return these mutilated wrecks to the world not as objects of horror and commiseration, but as men presentable, happy and fit to resume their places in society.

### Importance of Receiving Cases Early.

The lack of foresight and failure to create dental services generally began to make itself felt when the tardy arrivals (old cases) began to appear, sent to us for help, having passed beyond the general surgeons' sphere, cured of infection, but with mouths sewed up, faces distorted mastication rendered impossible, and with fractured parts not only displaced, but healed, ossified or ankylosed in abnormal positions, making double the work of restoration and necessitating either radical surgical intervention or long and tedious treatment, for which devices had to be made, taxing to the utmost professional skill and ingenuity.

As the opportunity and demand for service increased, offers of assistance were gladly accepted, first from local confreres. Dr. Hotz became a member of the staff in late October, giving half of his time. Professor Potter, of Harvard University, was with us for three months. At his special request, Dr. Darcissac became mobilized with us. Dr. Ortion was with us for six weeks. Since the middle of March Dr. Stuhl has given valuable assistance. The reputation and the character of the work attracted Dr. D. O. M. Le Cron and Dr. Roberts, of London, who contributed three months of skilled and faithful work to the service.

Then, in reply to requests from abroad, it was decided to give an opportunity to help to representatives of the various dental colleges in America. This decision was arrived at when it became a question of either limiting the number of "fracture cases" or of sending for help and converting the chapel into a second operating room. Your honorable Board then "recommended, in recognition of the services rendered by the department, to increase its capacity and set aside forty more beds for these special cases."

### Limit to Number of Fracture Cases.

Consideration of the other surgical departments, it might seem, has been the only reason for limiting the continued growth of this department, as material and assistance have at no time been lacking.

On July 1st, Professor Guilford, Dean of the Philadelphia Dental College, with Dr. Dudley Guilford and Dr. Wass, together with Dr. Speakman, Drs. Lane and Cooper, and Mr. Lenzer, of the University of Pennsylvania, arrived, soon followed by Dr. Russell, oral surgeon, from Philadelphia, and Dr. Robert Le Cron, of London.

It is my pleasure to report a universal interest and devotion to the work on the part of the entire staff, and to acknowledge most courteous consideration and co-operation of the general surgeons.

REPORT FROM SEPTEMBER 6, 1914, TO SEPTEMBER 1, 1915.

| | |
|---|---|
| Extractions | 2,658 |
| Amalgam Fillings | 1,345 |
| Cement Fillings | 627 |
| Gutta Percha Fillings | 247 |
| Arsenic Application | 192 |
| Roots filled | 399 |
| Pulps removed | 181 |
| Wire splints | 6 |
| Rubber splints | 14 |
| Metal splints | 88 |
| Bridge splints | 11 |
| Gold splint | 1 |
| Obturators | 2 |
| Angle arches | 4 |
| Pivots | 10 |
| Plates repaired | 9 |
| Total number of plates (vulcanite) 1,948 teeth. | 383 |

Cases of fractured maxillae:—

94 in hospital.
46 outside still coming for treatment.

———

140 under treatment.
104 finished.

———

| | |
|---|---|
| Total: 244 ................................ | 244 |
| Cleansing and Prophylactic treatments......... | 1,279 |
| Total number of cases treated......... | 1,523 |

Respectfully submitted,
G. B. Hayes,
*Chief Dental Surgeon.*

